# SHP-1: the next checkpoint target for cancer immunotherapy?

**DOI:** 10.1042/BST20150251

**Published:** 2016-04-11

**Authors:** H. Angharad Watson, Sophie Wehenkel, James Matthews, Ann Ager

**Affiliations:** * Systems Immunity University Research Institute and Division of Infection and Immunity, School of Medicine, Henry Wellcome Building, Cardiff University, Heath Park, Cardiff, CF14 4XN, U.K.

**Keywords:** adoptive cell transfer, checkpoint inhibitors, protein tyrosine phosphatase inhibition, SHP-1, tumour immunotherapy

## Abstract

The immense power of the immune system is harnessed in healthy individuals by a range of negative regulatory signals and checkpoints. Manipulating these checkpoints through inhibition has resulted in striking immune-mediated clearance of otherwise untreatable tumours and metastases; unfortunately, not all patients respond to treatment with the currently available inhibitors of cytotoxic T-lymphocyte-associated protein 4 (CTLA-4) and programmed cell death protein 1 (PD-1). Combinatorial studies using both anti-CTLA-4 and anti-PD-1 demonstrate synergistic effects of targeting multiple checkpoints, paving the way for other immune checkpoints to be targeted. Src homology 2 domain-containing protein tyrosine phosphatase 1 (SHP-1) is a widely expressed inhibitory protein tyrosine phosphatase (PTP). In T-cells, it is a negative regulator of antigen-dependent activation and proliferation. It is a cytosolic protein, and therefore not amenable to antibody-mediated therapies, but its role in activation and proliferation makes it an attractive target for genetic manipulation in adoptive transfer strategies, such as chimeric antigen receptor (CAR) T-cells. This review will discuss the potential value of SHP-1 inhibition in future tumour immunotherapy.

## Introduction

Immunotherapy has ushered in a new era in cancer treatment. Both the success of immune checkpoint inhibition strategies, and the limitations, which include non-responsiveness of some patients, as well as toxicity, has led to a search for new checkpoint targets. At the same time, the rise of cell-based immunotherapy, and an improved range of techniques for genetic modification, has expanded the range of possible targets to include intracellular checkpoints such as Src homology 2 domain-containing protein tyrosine phosphatase 1 (SHP-1). In this brief review, the potential of SHP-1 in the context of current immunotherapy strategies will be discussed.

## Checkpoint inhibition as an anti-tumour strategy

Until the start of the 21st century, all cancer treatment strategies focused on targeting and directly killing cancer cells. However, greater understanding of the regulation of T-lymphocytes in the late 1980s and 1990s led to an entirely new strategy for tumour treatment; exploiting T-cell regulatory molecules to ‘arm’ the immune system in order to clear tumours. The first of these checkpoint inhibitors to reach the clinic was an anti-cytotoxic T-lymphocyte-associated protein 4 (CTLA-4) antibody, ipilimumab, which first demonstrated effectiveness in the treatment of melanoma in 2008 [1,2]. This was closely followed by therapies targeting programmed death receptor-1 (PD-1) [3], the ligand for which, programmed death ligand-1 (PD-L1), is widely expressed by tumour cells [4,5]. These strategies have been recently and comprehensively reviewed elsewhere [6,7], so will not be discussed in further detail here; but their importance in signalling a sea-change in cancer therapy should not be underestimated.

## Adoptive cell therapy

Although checkpoint inhibition seeks to improve the ability of endogenous T-cells to clear tumours, adoptive transfer can take one of two approaches; *ex-vivo* expansion of a patients' own tumour-infiltrating lymphocytes (TILs) which are then infused back into the patient [8], or generation of T-cells genetically modified to target the tumour, either through introduction of tumour-specific T-cell receptors (TCRs) or chimeric antigen receptors (CARs) [9,10], which replace the antigen recognition domain of a TCR with the epitope binding moiety of an antibody [11]. The former strategy suffers from the same limitation as checkpoint inhibition; it relies upon the existence of endogenous T-cells specific for the tumour. As tumours develop from normal tissue, many of their antigens are recognized as ‘self’, and those that are not are generally poorly immunogenic [12]. Mutations during tumorigenesis give rise to ‘neoantigens’; novel antigens that can be targeted by the immune system [13]. Incidence of neoantigens is associated with improved response to checkpoint inhibitor therapy [14]. Unfortunately, neoantigens are not equally distributed across cancer types [15], meaning that either checkpoint inhibition or adoptive transfer of endogenous TILs is unlikely to offer clinical benefit to patients with low-neoantigen malignancies, which include most haematological malignancies. In contrast, the greatest success to date with CAR-T-cell therapy has been with chronic lymphoid leukaemia, as circulating cancer cells may be targeted by their expression of CD19 [16]. Like any other cell-based therapy, CAR-T-cells are subject to suppression by the tumour microenvironment, and also carry the additional risk of on-target, off-tumour toxicity, including normal B-cells expressing CD19. To address these limitations, researchers are examining all aspects of CAR design, from receptor affinity [17] to adding additional properties to CAR-T-cells, such as cytokine production or release of neutralizing scFvs directed against checkpoint inhibitors in so-called ‘armoured CAR-T-cells’ [18].

## Src homology 2 domain-containing protein tyrosine phosphatase-1 in T-cells

SHP-1 [protein tyrosine phosphatase, non-receptor type 6 (PTPN6)] is expressed by all mature haematopoietic lineages and at low levels, in a different isoform, by endothelial cells [19]. There is 95% homology between human and mouse SHP-1, making it amenable for study in pre-clinical mouse models [20]. SHP-1 consists of three domains; the N-terminal Src homology-2 (SH2) domain, the C-terminal SH2 domain, and the C-terminal catalytic protein tyrosine phosphatase (PTP) domain [21]. The N-terminal SH2 domain is auto-inhibitory; binding to the PTP domain until the C-terminal SH2 domain binds to a phosphopeptide ligand, allowing a conformational change and the release of autoinhibition [21]. Maximal phosphatase activity is achieved only when both SH2 domains are engaged [22]. Given this requirement, it is likely that SHP-1 interacts with proteins of the inhibitory-receptor superfamily (IRS) containing immunoreceptor tyrosine-based inhibitory motifs (ITIMs) (I/V/LxYxxL/V) within their cytoplasmic tails [23]. It has been shown that SHP-1 constitutively interacts with ITIM-containing leucocyte-associated immunoglobulin receptor-1 (LAIR-1) [24], what is less clear is whether it directly interacts with PD-1, which also contains a cytoplasmic ITIM domain [25]. Studies in human CD4 T-cells and JURKAT cells have demonstrated co-immunoprecipitation of SHP-1 and PD-1 [26,27], however, a recent study in human CD8 T-cells found that SHP-1 and PD-1 acted independently to inhibit T-cell activation; with PD-1 preferentially inhibiting T-cells with the highest affinity TCRs, while SHP-1-mediated inhibition increased incrementally as TCR affinity increased [28]. Furthermore, only SHP-2 has been demonstrated to interact directly with PD-1 in activated T-cells [29]. CTLA-4 does not contain any ITIMs, but does have cytosolic tyrosines that could represent potential binding sites for SHP-1, however, although other PTPs have been shown to associate with these cytosolic tyrosines, there is no direct evidence for SHP-1 interaction with CTLA-4 [30]. To date, no combinatorial studies of SHP-1 inhibition together with PD-1 or CTLA-4 inhibition have been conducted, however, the studies discussed above, in particular the work by Hebeisen et al. [28], suggest that such combinations are more likely to be synergistic than redundant in their anti-tumour effects.

**Figure 1:**
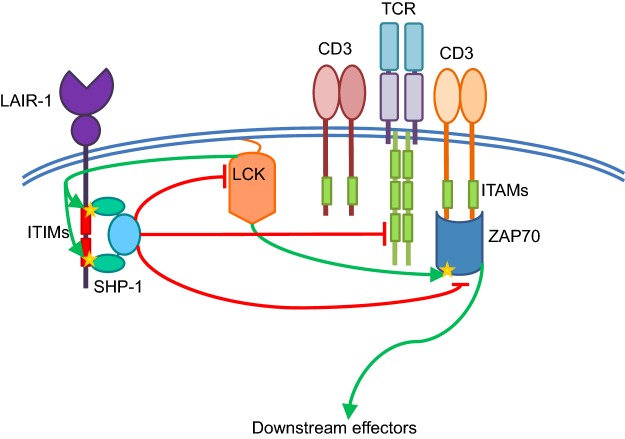
SHP-1 mediated inhibition of TCR signalling SHP-1 is constitutively associated with the inhibitory receptor LAIR-1, which, in turn, is constitutively phosphorylated by Lck [74], although SHP-1 may also be activated by other ITIM-containing inhibitory receptors. Activation of SHP-1 allows it to inhibit antigen-induced TCR signalling either through direct dephosphorylation of the TCRζ chain, or dephosphorylation of downstream adaptor proteins such as Lck and ZAP70. Activating phosphate groups are shown as stars.

**Figure 2:**
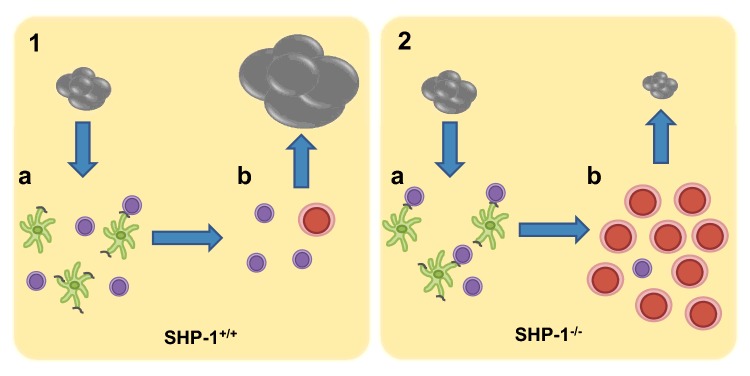
Lowered activation thresholds, increased duration of interaction with antigen presenting cells (green) and increased expansion of SHP-1^−/−^ CD8 T-cells are beneficial in tumour therapy (**1a**) Tumour antigens (grey) are low affinity and poorly immunogenic, and offer limited stimulation to naïve T-cells (purple). (**1b**) Low numbers of tumour specific effector T-cells (red) are insufficient to control tumour growth. (**2a**) SHP-1^−/−^ T-cells have lower activation thresholds, therefore can be stimulated by low-affinity antigens. (**2b**) In response to antigen stimulation SHP-1^−/−^ T-cells proliferate more than WT T-cells, leading to increased numbers of tumour specific effector T-cells, and predicted control of tumour growth.

Other than LAIR-1, little is known for certain about SHP-1 binding partners in T-cells, and there is similar debate regarding its substrates, although zeta-chain associated protein kinase 70 (Zap70) [31], lymphocyte-specific protein tyrosine kinase (Lck) [32], phosphoinositide 3-kinase (PI3K) [33], Vav [34] and TCRζ [35] are all strongly implicated [36] (Figure 1). However, the functional effect of SHP-1, or, rather, its absence, on T-cells is better understood. In the absence of SHP-1, CD8 T-cells form more stable and durable synapses with antigen presenting cells (APCs) [37]. This leads to reduced activation thresholds and increased proliferation [38], which is beneficial for any kind of adoptive transfer strategy for two reasons: firstly, numbers of T-cells available for transfer are often limited, especially where genetic modification is involved; and, secondly it is known that the balance of regulatory T-cells to effector T-cells is key in tumour progression [39], so any modification that can bias towards increased effector T-cells is likely to improve treatment efficacy (Figure 2). It is worth noting that SHP-1 has also been shown to be inhibitory to T regulatory cells [40], and therefore inhibition of SHP-1 in these cells leads to increased suppressor function. As with CD8 T-cells, this effect is attributed to increases in TCR–APC conjugate formation and duration. Specific deletion of SHP-1 in all CD4 T-cells via a floxed Shp1^fl/fl^ CD4-cre system in mice demonstrated a key role for SHP-1 in negatively regulating the responsiveness of CD4 T-cells to interleukin-4 signalling, and therefore maintenance of a TH1 phenotype [41]. Deletion of SHP-1 in other haematopoietic lineages in mouse models, such as B-cells, neutrophils and dendritic cells, is associated with a variety of pathologies [42–45], although SHP-1^−/−^ CD 8 T-cells have not been linked to any pathological effects, to date.

## A natural model

In 1965, a spontaneous recessive mutation was observed among the mice in Jackson Laboratories, and was given the name ‘motheaten’ due to the marked skin lesions observed on homozygous animals [46]. Motheaten mice die at 3–4 weeks of age, but in 1985 a new mutant mouse was described that had a mutation in the motheaten locus, but survived up to 9 weeks of age; this mouse was dubbed ‘motheaten viable’ [47]. It was not until 1993 that these mutations were associated with a haematopoietic phosphatase [48,49], later named SHP-1 by consensus. The motheaten mouse suffers a range of pathologies, including myeloid-driven skin lesions, interstitial pneumonitis (usually fatal), and a range of haematological abnormalities; polyclonal activation of B-cells, decreased NK cell activity, haemolytic anaemia, decreased dermal dendritic cells, as well as the previously described hyperproliferative T-cells [50,51]. The short lifespan of these animals and the range of multifactorial immunopathologies make them difficult to use effectively in the study of T-cell function. However, the extent of immune dysregulation in these animals indicates the importance of SHP-1 in the regulation of the immune system, and further suggests that specific targeting of SHP-1 in individual cell populations might be a safer approach in patients, rather than global inhibition, as in anti-CTLA-4 and anti-PD-1 therapies.

## SHP-1 abrogation in cancer therapy

A number of strategies to exploit the benefits of SHP-1 abrogation have been attempted to date. In pre-clinical studies, adoptive transfer of SHP-1 knockout T-cells has been shown to be beneficial in a model of leukaemia [52], whereas two phase I clinical trials have been taken place to assess the safety of using systemic treatment with sodium stibogluconate (SSG), a licensed treatment for leishmaniasis that is also an active-site inhibitor of SHP-1 and the related SHP-2, as a cancer therapy [53,54]. A further pre-clinical study has looked at developing a new, orally-bioavailable (SSG must be infused intravenously) SHP-1 inhibitor; a small-molecule, aromatic compound denoted as tyrosine phosphatase inhibitor 1 (TPI-1) by the authors [55]. In this study, TPI-1 was found to be ∼58 times as effective as SSG *in vitro*, and elicited an anti-tumour effect against 4-day established B16 melanomas *in vivo*, where SSG failed to have any effect. NSC-87877 is a small molecule competitive inhibitor of SHP-2, which is also inhibitory to SHP-1 [56] and is being explored as an anti-tumour agent, however this is due to its inhibitory effects on dual specificity protein phosphatase 26 (DUSP 26), which is overexpressed in neuroblastoma, rather than as a result of SHP-1 inhibition [57]. Suramin is another anti-parasitic agent that has been found to mediate active-site inhibition of a range of PTPs, and is therefore being investigated as an anti-tumour agent, however, its wide spectrum of target PTPs puts it beyond the scope of this review [58]. Historically, active-site-directed inhibitors of PTPs have been challenging due to the problem of creating cell membrane-permeable yet highly negatively charged compounds, however, recently, a cryptic allosteric inhibition site has been successfully targeted in SHP-2 [59], which represents a new strategy for PTP inhibition that might improve the clinical applicability of PTP inhibition.

In the study by Stromnes et al. [52], the authors used an Lck-driven cre to knockout floxed SHP-1 in mature T-cells. This system was used in preference to the SHP-1^null^ motheaten mouse, in order to avoid any confounding influence of other aberrantly activated SHP-1^null^ immune cells [60] on the maturation of the T-cells. In order to mimic clinical adoptive transfer strategies, T-cells were subject to three rounds of *in vitro* antigen stimulation prior to transfer. Although this system might appear to fail to take advantage of the increased antigen-dependent proliferation of naive SHP-1^null^ T-cells described by Sathish et al. [37,61], the authors observed increased proliferation of transferred effector T-cells in response to tumour *in vivo*, reduced apoptosis and improved survival of SHP-1^−/−^ T-cells, and, ultimately improved clearance of leukaemia. This demonstrates that abrogation of SHP-1 is beneficial in effector T-cells, not just in naive T-cells, and therefore knocking out SHP-1 in *in vitro*-activated, genetically modified T-cells would still add value to adoptive transfer strategies.

To date, although carried out in cancer patients, clinical trials of small-molecule SHP-1 inhibitors remain restricted to phase I dosing studies, and therefore anti-tumour effects, although measured, were not the primary purpose of the studies. In the event, no clinically measurable anti-tumour effects were observed in either study [53,54]. Although not the purpose, this is disappointing and does bring into question the effectiveness of SSG administration as an anti-cancer strategy. No phase II studies of small-molecule SHP-1 inhibition have been completed. Evaluation of toxicity of SSG was somewhat limited in both studies due to the combination of SSG with interferon and/or chemotherapy, and therefore where severe and/or life threatening adverse effects were observed (in up to 68% of patients), it was difficult to establish which treatment was responsible. Dose-limiting toxicities observed included pancreatitis, bone marrow suppression, fatigue, lipase elevation and gastrointestinal upset. Not observed was the fatal cardiac toxicity seen in 5–7% of leishmaniasis patients treated with SSG [62]. Both studies concluded that SSG treatment was well tolerated.

Interestingly, especially when considering global SHP-1 inhibition with agents such as SSG or TPI-1, SHP-1 expression is altered in a range of malignancies; up-regulated in breast and ovarian cancers [63,64], and gene-silenced in lymphomas, leukaemias and colorectal cancers [65–67].

## Future strategies

The disappointing performance of SSG/TPI-1 as an anti-cancer agent in both the pre-clinical and clinical studies described above suggests that the adoptive transfer approach of Stromnes et al. [52] might be the most promising avenue for exploitation of SHP-1 inhibition for tumour therapy. The cytosolic nature of SHP-1, and the difficulty in identifying inhibitors that will not target SHP-2 and other PTPs, means that genetic manipulation would be the best strategy for translational studies. There are currently a range of different techniques available for genetic manipulation that have been utilized in various adoptive transfer and CAR-T-cell approaches. A recent study used zinc finger nucleases via RNA electroporation to knockout PD-1 in TILs on a clinical scale in order to treat metastatic melanoma [68], however, limited success meant only *in vitro* evaluation of the modified cells was possible. In our own lab, we are currently investigating a zinc finger nuclease approach for ablating SHP-1 in human CD8 T-cells for tumour therapy. In the past, lenti- and retrovirally mediated gene transfer strategies have been popular, but difficulties with transduction of T-cells has led to electroporation of either DNA or RNA becoming the method of choice. CAR-T-cell therapies have optimized a number of genetic modification approaches, including the Sleeping Beauty transposon system [69], clustered regularly interspaced short palindromic repeats (CRISPR) [70] and transcription activator-like effector nucleases (TALEN) [71]. These approaches are reviewed in more detail elsewhere [72,73]. However, the range of clinically applicable gene transfer techniques available today mean that the additional knockout of a molecule like SHP-1 from T-cells already undergoing genetic modification becomes a much more straightforward proposition, making it more likely that the beneficial anti-cancer properties of SHP-1^−/−^ T-cells can be exploited in the clinic in the near future.

## Abbreviations

CAR: chimeric antigen receptor
CTLA-4: cytotoxic T-lymphocyte-associated protein 4, CD152
LAIR-1: leucocyte-associated immunoglobulin receptor-1
Lck: lymphocyte-specific protein tyrosine kinase
PD-1: programmed cell death protein 1, CD279
PTP: protein tyrosine phosphatase
PTPN6: protein tyrosine phosphatase, non-receptor type 6
SH2: Src homology 2 domain
SHP-1: Src homology 2 domain-containing protein tyrosine phosphatase 1, PTPN6
SSG: sodium stibogluconate
TALEN: transcription activator-like effector nucleases
TCR: T-cell receptor
TIL: tumour-infiltrating lymphocyte
Zap70: zeta-chain associated protein kinase 70
